# Evaluation of Knowledge, Awareness, and Occurrence of Dental Injuries in Participant Children during Sports in New Delhi: A Pilot Study

**DOI:** 10.5005/jp-journals-10005-1468

**Published:** 2017-02-27

**Authors:** Mridula Goswami, Puneet Kumar, Urvashi Bhushan

**Affiliations:** 1 Professor and Head, Department of Pedodontics and Preventive Dentistry, Maulana Azad Institute of Dental Sciences, New Delhi, India; 2 Postgraduate Student, Department of Pedodontics and Preventive Dentistry, Maulana Azad Institute of Dental Sciences, New Delhi, India; 3 Senior Resident, Department of Pedodontics and Preventive Dentistry, Maulana Azad Institute of Dental Sciences, New Delhi, India

**Keywords:** Awareness, Mouthguards, Orofacial trauma, Sports injury.

## Abstract

**Aim:**

To evaluate the occurrence of dental injuries in children, the level of knowledge of the participants about preventive measures, and management of dental trauma during sports, in New Delhi.

**Materials and methods:**

A cross-sectional study was carried out among 450 children aged 6 to 16 years. A structured interviewer-guided questionnaire was used to determine the prevalence of oral injuries sustained during sport activities, the use of mouthguard as well as the athlete’s awareness regarding use of mouthguard. The respondents consisted of 313 males (69.6%) and 137 females (30.4%) with a mean age of 12.6 years.

**Results:**

Out of the total participants, 27 (6%) had chipping or fracture of teeth, 25 (5.6%) had soft-tissue laceration, 24 (5.4%) had avulsion of teeth, and 18 (4%) had suffered fracture of jaw/bones. Out of the total participants, 263 (58.4%) knew that it was possible to replant the teeth and 187 (41.6%) did not know that it was possible to replant the teeth. Out of the total participants, 203 (45.1%) did not know what is the best time to put the teeth back in the mouth and 247 (54.9 %) answered in affirmative with variable answer. Of the total participants, 223 (49.6%) answered that they would carry avulsed tooth in water, 94 (20.8%) wrapped in cloth, 57 (12.6%) in mouth/saliva, 9 (2%) in Hanks’ balanced salt solution (HBSS), and 67 (14.8%) answered others. Of the total participants, 321 (71.3%) were aware that mouthguards prevent injury and 129 (28.7%) did not know about mouthguards. Out of the total participants, 94 (20.9%) used mouthguards and 356 (79.1%) had never used mouthguards.

**Conclusion:**

Level of awareness and knowledge about sports-related orofacial injury is very poor among children in New Delhi. Education on prevention of orofacial trauma should be given to the coaches and children. Wearing of mouthguards during sport activities should be compulsory during practice and competition events.

**How to cite this article:** Goswami M, Kumar P, Bhushan U. Evaluation of Knowledge, Awareness, and Occurrence of Dental Injuries in Participant Children during Sports in New Delhi: A Pilot Study. Int J Clin Pediatr Dent 2017;10(4):373-378.

## INTRODUCTION

Infants, children, and adolescents are engaged in recreational and competitive sports activities for both physical and psychological well-being. Unfortunately, participating in sports activities is at risk of sustaining trauma to the oral hard and soft tissues, such as chipped, luxated or avulsed teeth, maxillary or mandibular fractures, lip lacerations and other injuries to the gingiva, tongue, or mucosa.^1-4^ Dental injuries are the most common type of orofacial injury sustained during participation in sports.^5^ Other etiological agents which account for trauma are fall and road traffic accidents.^6-9^ Various studies have found that sport-related injuries account for 10 to 36% of injuries from all causes.^8-15^

With the increased popularity of contact sports and encouragement to participate at an early age, the role of the dental profession in relation to prevention of dental and other orofacial sporting injuries has become more important. In view of this, children, coaches, trainers, parents, and members of the dental community should be aware of how individuals who participate in sporting activities are at risk for dental trauma. The common orofa-cial sports-related injuries include soft-tissue injuries and hard-tissue injuries. Hard-tissue injuries include those injuries which involve injuries of teeth and facial bones, such as tooth intrusions, luxations, crown and/or root fractures, complete avulsions, and dentofacial fractures. Sports dentistry had its origins in 1980s and it encompasses the recognition of the injury-prone dentition and expertise in immediate management of dental injuries.

Dental injury among contact sport participants commonly involves the upper front teeth, which may chip, fracture, loosen, or be avulsed. This injury may be devastating, affecting appearance, speech, and the ability to eat.^8^ Very few Indian-based epidemiological information exists about the incidence of dental injury in sports.

Moreover, despite national medical and dental groups recommending the use of professionally fitted headgears and mouthguards to minimize head, facial, and dental injury, it has not been very popular among children in India. Mouthguards, also referred to as gum shields or mouth protectors, have long been promoted as a way to reduce the incidence of orofacial injuries and concus-sions.^16-19^ Mouthguards are hypothesized to reduce the likelihood of orofacial injuries through several mechanisms. Firstly, they may prevent fracture or discoloration of teeth by separating the mandibular and maxillary teeth and absorbing and redistributing shock during direct forceful impacts. Secondly, mouthguards may protect against mandibular bone fractures by absorbing shock, redistributing shock, and/or stabilizing the mandible during traumatic jaw closure. Thirdly, the mouthpiece may reduce laceration and bruising of soft tissue by separating the teeth from soft tissue, thus cushioning and distributing the force of impacts. In spite of high occurrence of trauma during sports, sports dentistry has been a neglected field all over the world and also in India. This study was done to evaluate the knowledge, awareness, and occurrence of dental injuries in participant children during sports in New Delhi.

## MATERIALS AND METHODS

**Graph 1:**
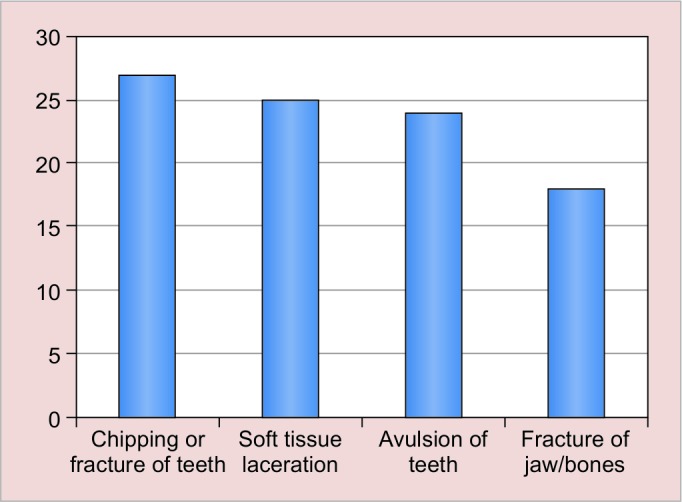
Type of injuries sustained

This cross-sectional study was carried out from May to June 2013 at two sports camps in New Delhi after taking prior permission from the authorities. The children who participated played different sports, i.e., football, volleyball, basketball, athletics, badminton, cricket, skating, boxing, tennis, table tennis, judo, swimming, gymnastic, and squash. All children who were aged 16 years and below, registered with the camps, and able to understand English/Hindi language were invited to participate in the study. A structured, interviewer-guided questionnaire was developed for data collection (Graph 1). The questionnaire was validated by a panel of experts and pretested on 25 children participating in sports activities for their understanding of the language. The questionnaire contained 20 items, including type of sports that children are involved, period of time they had been practicing that sport, whether any dental and soft-tissues injuries incurred; specifically loosening of teeth, fracture of teeth, broken bones, bruises on the face and lacerations on lips, tongue or cheeks while participating in sports activities. Participants were asked whether they knew that it was possible to reimplant the avulsed tooth and also the extraoral time within which it is possible to reimplant the teeth. Participants were asked whether they use mouthguard during sport activities and if not, state the reasons of not using and whether they believe mouthguard can prevent dental injury. The collected data were statistically analyzed.

## RESULTS

**Fig. 1:**
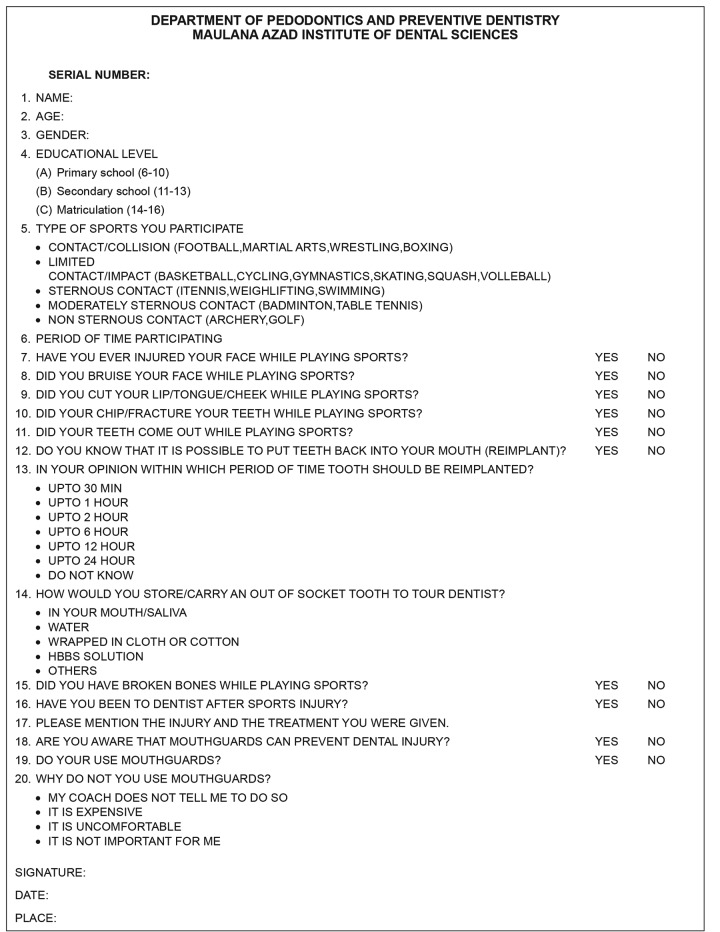
Questionnaire

A total of 450 children from the various sports camps participated in the study. Male children (69.5%) were accounted more than female children (30.5%). Their age ranged from 6 to 16 years and mean age was 12.6 years. Out of the total participants, 121 (26.8%) were in primary school level, 252 (56%) were in secondary school level, and 77 (17.1%) were from matriculation level. Most of the participants were secondary school students. The mean duration of involvement in the particular sports was 4 years. Children participated in various sports activity ranging from football, volleyball, basketball, athletics, badminton, cricket, skating, boxing, tennis, table tennis, judo, swimming, gymnastic, and squash. The distribution of participants in various sports is given in Figure 1.

**Graph 2:**
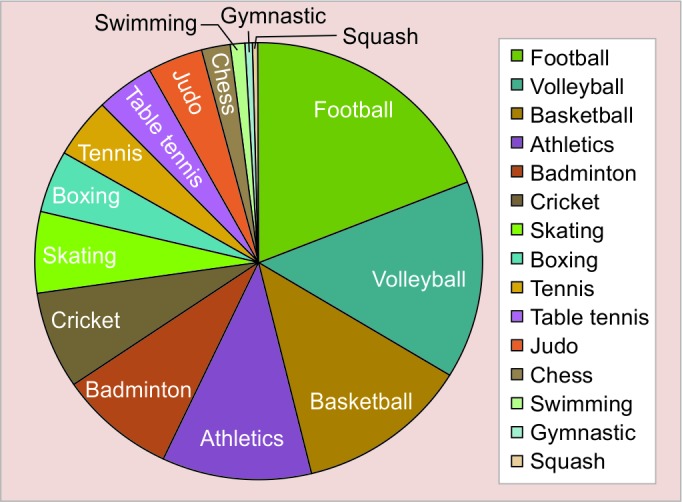
Distribution of participants in various sports

- Out of the total participants, 94 (20.9%) had sustained orofacial injury during sports and 356 (79.1%) had never sustained orofacial injury in any form. The type of injury sustained varied from chipping or fracture of teeth, soft-tissue laceration, avulsion of teeth to fracture of jaw and bones (Graph 2). Out of 94 participants, 27 subjects had experienced chipping or fracture of teeth, 25 had experienced soft-tissue laceration, 24 suffered from avulsion of teeth, and 18 had experienced fracture of jaw.
- Out of the total participants, 321 (71.3%) were aware that mouthguards prevent injury and 129 (28.7%) did not know about mouthguards but only 94 (20.9%). Most of the participants (48.6%) did not use mouth-guards during sports because they were not directed by their coach for using it, while others thought that it was not important (28.8%), uncomfortable (12.6%), or expensive (10.0%). Various reasons for not using mouthguards have been summarized in Table 1.

- Out of the total participants, 263 (58.4%) knew that it was possible to reimplant the teeth and 187 (41.6%) did not know that it was possible to replant the teeth. Most of the children (45.1%) were unaware of the time duration during which teeth could be reimplanted, while others responded with variable timing about reimplantation of tooth. The data have been summarized in Table 2.
- Various medium can be used for carrying an avulsed tooth. Almost 50% of the participants thought water was the best suited medium for carrying avulsed teeth to dentist, while only 2% knew that HBSS was the best suited medium. The data are summarized in Table 3.

**Table 1:** **Table 1:** Reasons for not using mouthguard during sports

*Reason*		*Number of participants*		*Percentage*	
My coach does not ask me to wear		219		48.6	
It is not important for me		130		28.8	
It is uncomfortable		57		12.6	
It is expensive		44		10.0	

**Table 2:** **Table 2:** Time duration for teeth to be reimplanted

*Time*		*Number of participants*		*Percentage*	
Within 30 minutes		56		12.6	
Within 1 hour		73		16.4	
Within 2 hours		16		3.6	
Within 6 hours		23		5.1	
Within 12 hours		6		1.3	
Within 24 hours		73		16.4	
Do not know		203		45.1	

**Table 3:** **Table 3:** Medium for carrying the avulsed tooth in case of orofacial injury

*Medium*		*Number of participants*		*Percentage out of total participants*	
Water		223		49.6	
Wrapped in cloth		94		20.8	
Mouth/saliva		57		12.6	
HBSS solution		9		2.0	
Others		67		14.8	

## DISCUSSION

Sports activities are unfortunately associated with injury risks that include orofacial soft- and hard-tissue trauma and such accidents often have life-long consequences. The present study reveals 20.9% of athletes had experienced one or more form of orofacial injury during sport activities. This result corroborate with studies done by Tulunoglu and Ozbek^12^ and Persic et al,^13^ where 22.3 and 20.4% of the participants reported to have experienced oral injuries respectively. The prevalence of dental trauma among Pan American games athletes was 49.6%, where 63.6% of them were sustained during training or competition.^20^ Sports-related dental injuries have accounted for high percentage among all types of traumatic injuries across the world.^20-22^

Among the sportsperson who had injuries in this study, chipping/fracture of teeth occurred most frequently (28.7%) followed by laceration on face (26.6%), whereas for hard-tissue injuries namely loosening of teeth, fracture teeth, and facial bone fracture were relatively lesser. Commonly, these injuries incurred in children when they fall on their face or they are hit by hard objects from sports equipments and collision between players.^23^

The risk of oral injuries during performing sports and exercise activities can be reduced substantially by using mouthguards.^24^ Mouthguards offer protection by separating the cheeks and lips from the teeth, making users less susceptible to soft-tissue laceration and preventing opposing arches from traumatic contact, and these protective devices provide a resilient, protective surface to distribute and dissipate transmitted forces on impact.^5^ Regarding the usage and awareness of mouthguard, 20.9% of the athletes in the present study were utilizing it. However, 71.3% of those athletes knew that a mouth-guard can prevent orofacial injuries. Athletes not wearing mouthguards are also common in many other countries. Various studies have shown that although the participants were well aware of importance of mouthguards, very few were actually using them.^1,9,14,15^ These findings support that knowledge alone on mouthguard use does not ensure its utilization. Collaborations between sports authorities and dental professionals are recommended to increase the awareness and promote the use of mouthguards among athletes and coaches. Regulatory bodies should also maintain regulations requiring all athletes to wear mouthguards.

Furthermore, athletes should be informed that the physical impacts of having injuries far exceed the costs of purchasing and the inconvenience of wearing a mouth-guard. Most common reason why athletes (48.6%) did not wear mouthguards was that their coaches did not urge them to wear. It is important to inform athletes and coaches about the need of mouthguards in both contact and noncontact sports. The present study revealed that cost is not a factor in not wearing a mouthguard, but lack of consideration on the importance of mouthguard in preventing oral injury is the main reason for not wearing a mouthguard. The fear of discomfort was the reason given by 12.6% of athletes in the present study. Resistance for wearing of mouthguard might be due to discomfort like interference with breathing and speech, and the effect on the players’ image.^25^

The state of knowledge among the individuals questioned about replantation of avulsed teeth was another aspect of our study to assess the level of information on emergency management of dental trauma. In the present study, 263 out of 450 (58.4%) individuals were aware that it was possible to reimplant the avulsed tooth. The results of a study showed 65 of the 112 (58.03%) interviewees were aware of the fact that an avulsed tooth can be replanted.^26^ Another study showed that only 34.7% of individuals were aware about reimplantation of teeth.^27^ Most of the participants (45.1%) in the study were unaware about the time duration in which the avulsed teeth could be reimplanted and the best suited medium for carrying avulsed teeth to dentist. Other studies have shown that 31.6% individuals had no idea about time duration in which avulsed teeth could be reimplanted and only 8.3% knew that avulsed teeth should be carried in liquid medium (milk, water, saliva).^27,28^ This result may be considered unsatisfactory because the high subsequent cost (which those questioned were unaware of) can be substantially decreased through a physiological tooth rescue. This is one of the important results of this study which draws attention to the lack and importance of education about dental trauma.

## CONCLUSION

It can be concluded from the present study that:

- A high percentage of participants suffered from orofacial injury during sports. The type of injury sustained varied from chipping or fracture of teeth, soft-tissue laceration, avulsion of teeth to fracture of jaw and bones.
- Most of the participants were aware about the role of mouthguards in prevention of injury, but majority of them did not use mouthguards because of lack of motivation from coaches.
- Almost 60% of participants knew that it was possible to reimplant the avulsed tooth but were unaware of the time duration during which teeth could be reim-planted.
- There was clear lack of knowledge regarding the medium for carrying avulsed teeth. Most of the participants thought that water was the best suited medium for carrying avulsed teeth to a dentist and only 2% knew about HBSS as a medium for carrying the tooth.

## ROLE OF PEDODONTISTS IN SPORTS-RELATED TRAUMA AND SUGGESTIONS

It is a matter of bit dismay that level of awareness about sports-related injuries is very poor among children even in national capital region of New Delhi in spite of better resources. Consequences of orofacial trauma for children and their families are substantial because of potential of pain, psychological, and economic implications. Children with untreated trauma to permanent teeth exhibit greater impacts on their daily living than those without having injury. The frequency of dental trauma is significantly higher for children with increased overjet and inadequate lip coverage. A dental professional may be able to modify these risk factors at an early stage. Since most of the children start participating in sports at an early age, sports dentistry should be included in educational curriculum at the level of secondary school. Pedodontists in collaboration with pediatricians should counsel parents about prevention and management of trauma due to sports-related dental injuries. Moreover, the lack of knowledge among coaches about the lack of various preventive tools in children makes them more prone to trauma. This has to be taken care of by organization of various educational programs. Knowledge imbibed early goes long way and thereby will help children to lead healthy life away from any disfigurement. Healthy children lay the foundation of healthy nation. We should recognize the prevalence of sports-related orofacial injuries in our nation’s youth and the need for its prevention.
